# Two-Year Clinical and Functional Outcomes of an Asian Cohort at Ultra-High Risk of Psychosis

**DOI:** 10.3389/fpsyt.2018.00758

**Published:** 2019-01-25

**Authors:** Chun Ting Chan, Edimansyah Abdin, Mythily Subramaniam, Sarah Ann Tay, Lay Keow Lim, Swapna Verma

**Affiliations:** ^1^Institute of Mental Health, Singapore, Singapore; ^2^Duke-NUS Medical School, Singapore, Singapore

**Keywords:** psychosis, ultra-high risk for psychosis, schizophrenia, outcome, treatment

## Abstract

**Background:** To determine the 2-year clinical and functional outcomes of an Asian cohort at ultra-high risk (UHR) of psychosis.

**Method:** This was a longitudinal study with a follow-up period of 2 years on 255 help-seeking adolescents and young adults at UHR of psychosis managed by a multi-disciplinary mental health team in Singapore. Clients received case management, psychosocial, and pharmacological treatment as appropriate. Data comprising symptom and functional outcomes were collected over the observation period by trained clinicians and psychiatrists.

**Results:** The 2-year psychosis transition rate was 16.9%, with a median time to transition of 168 days. After 2 years, 14.5% of the subjects had persistent at-risk symptoms while 7.5% developed other non-psychotic psychiatric disorders. 38.4% of the cohort had recovered and was discharged from mental health services. The entire cohort's functioning improved as reflected by an increase in the score of the Social and Occupational Functioning Assessment Scale during the follow-up period. Predictors to psychosis transition included low education level, baseline unemployment, a history of violence, and brief limited intermittent psychotic symptoms, while male gender predicted the persistence of UHR state, or the development of non-psychotic disorders.

**Conclusion:** Use of the current UHR criteria allows us to identify individuals who are at imminent risk of developing not just psychosis, but also those who may develop other mental health disorders. Future research should include identifying the needs of those who do not transition to psychosis, while continuing to refine on ways to improve the UHR prediction algorithm for psychosis.

## Introduction

Schizophrenia and related psychotic disorders impose significant social and economic burden on the patients and the society, with the World Health Organization estimating that the direct costs associated with schizophrenia to be about 2% of total health care expenditure (1).

Detecting and managing persons at Ultra High-Risk (UHR) for psychosis was identified as a potential way to recognize persons at increased risk of developing a psychotic disorder. It is presumed that with early identification and management, mental healthcare providers will be able to offer treatment to prevent the development of mental health disorders that may follow the prodromal phase.

However, there are significant variations in the psychosis transition rates reported across studies (2–8) which may be affected by factors such as study design, subject characteristics and follow-up duration. It also appears that the psychosis transition rate has been in the decline over the years (7, 9). Regardless, the common finding is that majority of UHR individuals do not develop a psychotic disorder (6, 10–14). This has important implications regarding patient education, treatment provision, and service planning.

Singapore is an island nation in South-East Asia with a population of 5.61 million persons (2017). This is a naturalistic study reporting on the 2-year symptom and functional outcomes of 255 help-seeking UHR individuals in Singapore. These individuals were managed by a multi-disciplinary team under the Support for Wellness Achievement Program (SWAP) which was established in 2008 and is based in the Institute of Mental Health, the only tertiary psychiatric hospital in Singapore. SWAP provides a comprehensive and integrated management program for UHR individuals aged between 16 and 30. Suitable patients are managed by the healthcare team for a maximum of 2 years. The period of care varies depending on the need and desire of the young persons and their families. Our multi-disciplinary team includes psychiatrists, case managers, psychologists, social workers, and occupational therapists. Details of the SWAP service have been described in an earlier article by Rao et al. (15)

## Methods

### Sample

This study included individuals accepted into SWAP between January 2008 and June 2014. They were assessed by trained psychiatrists, with their UHR status determined using the Comprehensive Assessment of At-Risk Mental State (CAARMS) scale at baseline. The subjects were aged between 16 and 30 years at intake and assessed to be in a prodromal state. Exclusion criteria included a previous episode of DSM-IV psychotic disorder, the presence of organic brain disease, serious developmental disorder, and physical and neurological illnesses that could cause psychosis.

All data was collected at the Institute of Mental Health and its satellite clinics in Singapore. Data was captured in a clinical database and anonymized before the analysis. The study protocol was approved by the Domain Specific Review Board of the National Healthcare Group.

### Assessment

Structured clinical and psychosocial assessments were conducted for patients at regular intervals. Diagnoses were confirmed by trained psychiatrists using the Structured Clinical Interviews for Diagnostic and Statistical Manual for Mental Disorders-4th Edition (SCID-I) (16). CAARMS was administered by trained case managers.

The level of functioning was measured using the Social and Occupational Functioning Assessment Scale (SOFAS) (17) and a survey of their vocational status.

SCID-I–The Structured Clinical Interview for DSM-IV Axis I Disorders (SCID-I) is a semi-structured interview for making the major DSM-IV Axis I diagnoses. The instrument is administered by a trained psychiatrist at baseline, 1 year, and 2 years.

SOFAS–The SOFAS is a scale that measures the individual's level of social and occupational functioning. It differs from the Global Assessment of Functioning in that it is not directly influenced by the overall severity of the individual's psychological symptoms. The SOFAS is used to rate current functioning and is rated on a scale of 0–100, which is done at baseline, 6 months, 1 year, and 2 years.

CAARMS–The Comprehensive Assessment of At-Risk Mental State (CAARMS) is a semi structured interview used to evaluate if an individual meets the UHR criteria. The positive symptom subscale was used, which assesses four symptom domains: unusual thought content, non-bizarre ideas, perceptual abnormalities, and disorganized speech. Each symptom was rated for the maximum intensity, frequency and duration, pattern, and related distress over the past 1 year. The 3 main criteria for UHR include the presence of (1) Brief Limited Intermittent Psychosis (BLIPS, with history of psychotic symptoms that resolved spontaneously within 1 week) (2) Attenuated Psychosis Syndrome (APS, having experienced subthreshold psychotic symptoms) or (3) Vulnerability group (Functional decline in a person with first degree family member suffering from psychosis). CAARMS is a widely used instrument in both Asian and Western centers (12, 18–20). CAARMS was done by trained case managers in person or by phone, and was administered at baseline, 1 year, and 2 years.

Violence was measured using self-reported information and family report. A positive answer from either the subject or their family was treated as positive for violence. These data were collected at baseline, 6 months, 1 year, and 2 years.

All measures were administered by trained clinicians. Clinical consensus was reached between psychiatrists in the study team if necessary.

### Outcomes

The primary outcome of the study was the transition to a primary psychotic disorder over the 2-year follow-up period. Secondary outcomes include the persistence of UHR state, the development of a non-psychotic psychiatric disorder, and the level of functioning at 2 years.

### Statistical Analysis

All statistical analyses were performed using STATA version 13. Descriptive statistics were computed for the basic demographic and clinical variables. Mean and standard deviations (SD) were calculated for continuous variables and frequencies and percentages for categorical variables. Differences between variables at baseline and last visits at 24 months were tested by paired *t*-test and Wilcoxon signed-rank test for normal and non-normal continuous variable whenever appropriate. Cox proportional hazards regression model was used to identify variables associated with conversion to psychosis. Multinomial logistic regression analyses were also used to predict persistence of ARMS and the development of psychotic disorder at year 2 follow-up. Level of significance was set at *p* < 0.05.

## Results

### Participants

A total of 343 patients were accepted into SWAP during the study period. Data from 255 patients was available for baseline analysis. The sample consisted of 173 males (67.8%) and 82 females (32.2%) with a mean age of 20.8 years (SD 3.3). There were 199 (78.0%) Chinese, 28 (11.0%) Malays, 23 (9.0%) Indians with the rest (2%) being Eurasians or others (Table 1). The study population was reflective of the racial distribution of the general population in Singapore (21).

**Table 1:** Baseline sociodemographic and clinical characteristics of the sample.

	**Mean**	**SD**
Age, year	20.8	3.3
	n	%
**GENDER**		
Male	173	67.8
Female	82	32.2
**RACE**		
Chinese	199	78.0
Malay	28	11.0
Indian	23	9.0
Others	5	2
**MARITAL STATUS**		
Single/Never married	246	97.2
Married	6	2.4
Separated	1	0.4
**EDUCATION**		
Primary and below	27	10.6
Secondary	115	45.3
Tertiary	112	44.1
**EMPLOYMENT STATUS**		
Employed	113	44.3
Unemployed	25	10.1
Economically inactive	110	44.3
**CAARMS GROUP**		
CAARMS-APS^a^ (%)	153	60.0
CAARMS-Vulnerable^b^ (%)	54	21.2
CAARMS-BLIPS^c^ (%)	7	2.7
Current smoker	47	18.7
Past suicide attempt at baseline	30	11.8
Past aggression or violence	71	28.0
1st degree family history of psychiatric illness	95	37.9
Past contact with the police	24	9.5

CAARMS, The Comprehensive Assessment of At-Risk Mental State;

a CAARMS-Vulnerable, Vulnerable group;

b CAARMS-APS, Attenuated psychotic symptom group;

c *CAARMS-BLIPS, Brief limited intermittent psychotic symptoms group*.

### Symptom Outcomes

At baseline, 153 (60.0%) fulfilled the criteria for APS, 54 (21.2%) for the vulnerable group and 7 (2.7%) for BLIPS. The remaining patients (16.1%) either did not fall into any specific subgroup but were determined to be in a prodromal state based on clinical decision or they fulfilled the criteria for more than 1 UHR group.

**Figure 1:**
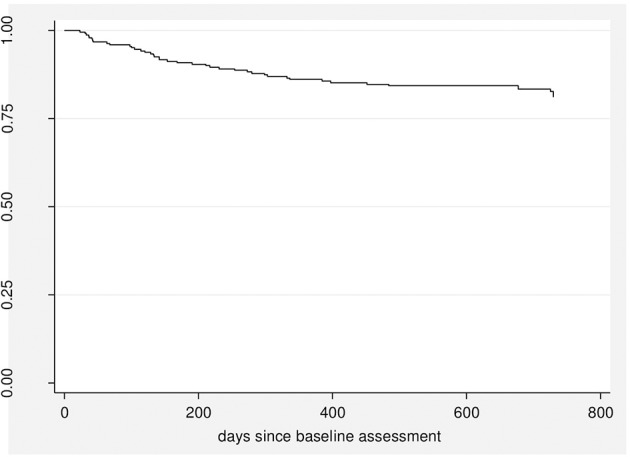
Kaplan-Meier estimates of the risk of developing psychotic disorder.

Over the 2-year follow-up period, 43 patients (16.9%) developed a psychotic disorder with a median time to transition of 168 days (Figure 1).

Thirty-seven patients (14.5%) continued to meet the criteria for UHR at 2 years. Nineteen (7.5%) required psychiatric care with other services but did not develop a psychotic disorder. Ninety-eight patients (38.4%) were discharged without the need for further psychiatric follow-up. One patient (0.4%) had defaulted during the follow-up period, and the 2-year data was not available for 56 (22.0%) of the patients.

### Predictors of Transition to Psychosis, Persistence of UHR Characteristics and Other Psychiatric Symptoms

Using the Cox regression model, a primary or lower education level (<6 years of formal education) (*p* = 0.047), the presence of history of violence (*p* = 0.003), unemployment at baseline (vs. employed) (*p* = 0.004), and BLIPS (*p* = 0.018) predicted the development of a psychotic disorder (Table 2).

**Table 2:** Predictors of transition to psychosis.

	**Hazard risk**	**95% confidence interval**		***P*-value**
Age	1.1	1.0	1.2	0.180
**GENDER**				
Male	Reference.			
Female	1.4	0.7	2.9	0.377
**RACE**				
Chinese	Reference			
Malay	0.8	0.3	2.2	0.643
Indian	.	.	.	.
Others	.	.	.	.
**MARITAL STATUS**				
Never married	Reference			
Single	3.2	0.5	20.0	0.213
Separated	.	.	.	.
Education				
Primary or lower	2.6	1.0	6.6	0.047
Secondary	Reference			
Tertiary	1.0	0.4	2.3	1.000
**EMPLOYMENT STATUS**				
Employed	Reference			
Unemployment	4.2	1.6	10.7	0.003
Student	1.2	0.5	2.8	0.683
**FAMILY HISTORY WITH MENTAL ILLNESS**				
No	Reference			
Yes	1.7	0.8	3.7	0.153
**SUICIDE ATTEMPT**				
No	Reference			
Yes	0.6	0.2	2.0	0.393
**VIOLENCE**				
No	Reference			
Yes	2.9	1.5	5.8	0.002
**POLICE CONTACT**				
No	Reference			
Yes	1.6	0.5	5.0	0.420
**CAARMS-VULNERABLE^a^**				
No	Reference			
Yes	0.6	0.2	1.4	0.202
**CAARMS-APS^b^**				
No	Reference			
Yes	1.1	0.6	2.2	0.787
**CAARMS-BLIPS^c^**				
No	Reference			
Yes	6.5	1.4	30.6	0.018

*. = Not estimated due to small sample size*.

CAARMS, The Comprehensive Assessment of At-Risk Mental State;

a CAARMS-Vulnerable, Vulnerable group;

b CAARMS-APS, Attenuated psychotic symptom group;

c *CAARMS-BLIPS, Brief limited intermittent psychotic symptoms group*.

Further comparisons between subjects who had developed psychotic disorders, persistence of UHR or developed non-psychotic psychiatric disorders, and discharged without the need for further psychiatric follow-up using the multinomial logistic regression model, we found that male gender (vs. female) (*p* = 0.024) was significantly more likely to have persistent UHR or the development of a non-psychotic psychiatric disorder than discharged without the need for further psychiatric follow-up (Table 3).

**Table 3:** Difference in sociodemographic and clinical characteristics between the three groups (transitioned, persistent ARMS / other disorders and recovered).

	**Persistence of ARMS features vs. Recovered**				**Developed psychotic disorders vs. Recovered**			
	**Odds ratio**	**95% confidence interval**		***P*-value**	**Odds ratio**	**95% confidence interval**		***P*-value**
Age	1.02	0.9	1.2	0.806	1.1	0.9	1.3	0.156
**GENDER**								
Male	Reference.				Reference.			
Female	0.3	0.1	0.8	0.024	0.9	0.4	2.1	0.747
**RACE**								
Chinese	Reference					Reference		
Malay	1.2	0.3	4.2	0.793	0.9	0.3	2.9	0.776
Indian	1.4	0.3	6.3	0.643	.	.	.	.
Others	1.4	0.2	15.5	0.78	.	.	.	.
**MARITAL STATUS**								
Single/Never married	Reference					Reference		
Married	8.1	0.3	225.2	0.216	6.8	0.4	115.0	0.184
**EDUCATION**								
Lower	.	.	.	.	2.8	0.9	9.3	0.087
Secondary	Reference					Reference		
Tertiary	0.8	0.3	2.2	0.728	1.03	0.4	2.7	0.951
**EMPLOYMENT STATUS**								
Employed	Reference					Reference		
Unemployment	3.1	0.7	13.1	0.133	7.5	2.1	27.2	0.002
Student	1.1	0.4	3.1	0.853	1.6	0.6	4.3	0.354
Smoking (Yes vs. No)	0.9	0.3	3.1	0.913	0.5	0.1	1.5	0.202
**FAMILY HISTORY WITH MENTAL ILLNESS**								
No	Reference					Reference		
Yes	1.4	0.5	3.8	0.453	1.7	0.7	4.3	0.238
**SUICIDE ATTEMPT**								
No	Reference					Reference		
Yes	2.0	0.6	6.6	0.244	0.8	0.2	4.1	0.990
**VIOLENCE**								
No	Reference					Reference		
Yes	1.1	0.4	3.0	0.843	2.9	1.2	6.9	0.014
**POLICE CONTACT**								
No	Reference					Reference		
Yes	0.1	0.01	1.5	0.104	1.01	0.2	4.1	0.990
**CAARMS-VULNERABLE^a^**								
No					Reference			
Yes	1.7	0.5	5.1	0.379	0.7	0.2	2.2	0.576
**CAARMS-APS^b^**								
No					Reference			
Yes	1.6	0.7	3.8	0.254	1.5	0.7	3.4	0.312
**CAARMS-BLIPS^c^**								
No					Reference			
Yes	.	.	.	.	6.0	0.8	46.7	0.088

*. = Not estimated due to small sample size*.

CAARMS, The Comprehensive Assessment of At-Risk Mental State;

a CAARMS-Vulnerable, Vulnerable group;

b CAARMS-APS, Attenuated psychotic symptom group;

c *CAARMS-BLIPS, Brief limited intermittent psychotic symptoms group*.

### Functional Recovery

The mean SOFAS score at baseline was 53.4 (*SD* = 10.1), indicating a serious impairment of functioning on initial presentation. Over the 2-year follow-up period, the cohort showed a significant improvement in SOFAS score (*p* < 0.001) which improved to 69.8 (*SD* = 13.4) at 2 years.

Whitehorn et al. defined functional recovery in a cohort of patients suffering from psychosis as SOFAS score >60 (22). Using this criterion, 70.1% of our patients were able to attain functional recovery at 2 years. The rates of functional recovery were slightly higher in those with persistent UHR (74.1%) than those with a non-psychotic psychiatric disorder (50%) and those who experienced full symptom remission (69.4%). The difference was however non-statistically significant (*p* = 0.737).

## Discussion

UHR states are conceptualized as clinical syndromes where individuals are at elevated risk of developing psychotic disorders. But studies have shown that UHR states can take on several possible clinical trajectories (11, 23–25), ranging from complete remission of all psychiatric symptoms, to the persistence of UHR states, to the development of psychotic, and non-psychotic psychiatric conditions. This highlights the importance of maintaining flexibility of mental health services in supporting young UHR individuals whose clinical symptoms may evolve over time.

Functional decline and the emergence of subthreshold psychiatric symptoms often precede the development of psychotic disorders such as schizophrenia (5, 26) (Addington and Heinssen, Prediction and prevention of psychosis in youth at clinical high risk., (27), and one of the functions of identifying UHR individuals in this “pre-illness” stage is so that evidence-based treatment can be instituted. This brings about the possibility of reducing the individual's risk of developing any psychiatric disorder, improving their mental well-being and functional outcomes.

### Psychosis Transition

This study examines the symptom and functional outcomes of help-seeking UHR individuals in an Asian population. The primary finding was that based on the current UHR criteria, the cumulative conversion rate to a primary psychotic disorder after 2 years was 16.9%, with a median duration to transition of about 5 months. In a meta-analysis involving 2,500 UHR individuals, Fusar-Poli et al. found a 29% transition rate (95% CI, 27.3–31.1%) within 31 months following first clinical presentation (23), and specifically, the transition risk at 24 months was 29.1% (23). This shows that transition rate in our cohort was low compared to that reported in other studies examining the short to medium term development of psychotic disorder in UHR individuals.

Transition rates vary between studies and factors influencing the observed rates include differences in study methodology, risk criteria, sample characteristics, duration of follow-up, and treatment. In addition, it has been observed that the rate of psychosis transition has reduced over the recent years. Yung et al. reported a reduction in the 12-month transition rate from 50 to 12% between 1995 and 2000 (9), which was not accounted for by differences in levels of pre-morbid functioning or severity of psychiatric symptoms. A possible explanation was the decrease in the duration of symptoms experienced by the patients before they received medical attention. This early detection allowed for the early identification of UHR individuals so that effective treatment could be instituted, reducing the rate of transition to psychosis.

The age of onset of psychotic disorders such as schizophrenia varies between studies. This variation can be attributed to the use of differing symptom criteria in determining the onset of the illness as well as the reliability of patient-reported or family-observed onset of behavioral changes (28, 29). The consensus on the age of onset of schizophrenia is that the incidence peaks before the age of 25 in men and between 25 and 35 for women (30). The mean age of our study population was 20.8 years. This suggests that a proportion of persons under our care may not have lived past the peak age of psychosis onset, contributing to the low transition rate. From a population perspective, illicit drug use is less common in Singapore (31, 32) and those who have an active substance use disorder have been excluded from SWAP and could have contributed to the low observed transition rate.

In addition, case management offered by SWAP may have been responsible for the low transition rate. In a double-blind, placebo-controlled trial, cognitive-behavioral case management (33) was found to be effective in reducing the 6-month conversion rate to psychosis. Our case managers are trained in providing psychological support while the team psychologists manage individuals requiring more in-depth structured therapies. This ensures that treatments with lower risk of adverse effects are made available to the UHR population, while at the same time providing benefits to those in need.

### Predictors of Transition

We found that significant predictors of transition were unemployment at baseline and having a history of violence. These factors are consistent with findings from previous research (5, 9, 23, 34, 35).

The relationship between violent behavior and psychotic disorders is complex and can be influenced by factors related to the illness as well as those associated with the person's socio-occupational state (36). Some examples of these factors are impulsivity, severity of the psychotic symptoms, unemployment, and housing status. We hypothesize that UHR individuals who are at the highest risk of transition exhibit elevated levels of impulsivity, a trait found in persons suffering from both early psychosis and those with longer duration of illness (37, 38). This impulsivity could have led to the increased rates of violence (37, 38) observed in the study.

It has been reported that those experiencing BLIPS are at increased risk of developing psychotic disorders (24, 39), which is consistent with findings from our study. This suggest that BLIPS may fall along the psychosis spectrum of disorders and that treatments, including the use of antipsychotics, should be considered in the earlier illness course for someone experiencing BLIPS. In our sample, we did not find those in the CAARMS—APS group were at elevated risk of transitioning to psychosis as compared to subjects in the CAARMS—Vulnerable group.

### Secondary Outcomes

The secondary aim of this study was to examine the outcomes of UHR individuals who did not develop a psychotic disorder. A significant proportion of our study population (24.0%) continued to experience persistent prodromal psychotic symptoms while 13.4% developed a non-psychotic psychiatric disorder requiring further attention. This highlights the fact that a significant proportion of UHR individuals are at risk of developing other psychiatric disorders or may continue to experience ongoing subthreshold symptoms. Hence treatment in these individuals should not merely focus on prevention of psychotic disorders but also address the myriad of other psychiatric symptoms and maladaptive coping that these individuals often exhibit.

Unemployment at baseline again predicted either the persistence of prodromal symptoms or the development of other non-psychotic psychiatric condition (40). UHR individuals often experience difficulties in their academic and occupational performance. This is consistent with the findings from our cohort where the mean baseline SOFAS score was 50.3, which indicates that many of them experienced serious challenges socio-occupational functioning. However, it is of interest to note that the proportion of individuals actively engaged in education or work remained high. This may be explained by the economic situation in Singapore.

Since 2003, Singapore has mandated compulsory primary education between the age of 6 and 15 years (41). In addition, there is a wide-range of options in higher education offered by the Singaporean government and private institutions. These would have contributed to the high proportion of the study cohort being engaged in education at baseline and at 2 years.

Furthermore, the unemployment rate in Singapore stands at a low of 2.2% in 2017, and there continues to be a large demand for both skilled and unskilled workers in the country. This is likely to be at least partially responsible for the low employment rate as seen in the study cohort.

There was significant improvement in SOFAS score to 69.8 after 2 years, reflecting an improvement in psychiatric symptoms and better psychosocial well-being from the multi-disciplinary services offered by SWAP. We did not identify any factor at baseline that could predict the 2-year functional outcomes of the cohort.

### Strengths and Weaknesses

The strengths of this study are the large sample size, a low dropout rate and the clearly defined criteria for UHR state from a single study site. The limitations include (1) A proportion of subjects who were accepted into SWAP during the recruitment time-frame did not have a baseline CAARMS assessment performed and were excluded from analysis. This may have included individuals with clinical characteristics not fitting the UHR state and which could have confounded the study's findings. (2) We did not capture the diagnosis individuals who developed a non-psychotic disorder. The information would have been useful in characterizing the clinical outcomes of UHR individuals. (3) Pharmacological and non-pharmacological treatment received by the subjects were not available in detail as the information was not universally collected and may have an influence on the subjects' symptom and functional outcomes.

## Conclusion

Research and ideas involving UHR states have evolved over time. The use of clinical criteria allows us to prospectively identify individuals at increased imminent risk for psychosis relative to the general population. Moreover, we know that a significant proportion of these individuals will have a persistence of prodromal symptoms and may go on to develop other psychiatric disorders. Many of them will experience significant functional impairments. These individuals are likely adolescents and young adults and should be monitored regularly. Adequately addressing the needs for these individuals through a multi-disciplinary management approach may allow us to delay or even prevent the onset of more serious mental health conditions. From the results of this study, we note that those with poorer baseline functioning are at increased risk of having persistent psychiatric symptoms, and mental health services should be tailored to the needs of these individuals.

The association between low education level and an increased rate of transition indicates that it is important for mental healthcare services to allocate increased resources and attention to young persons with lower academic achievements and/or are not employed on entry into mental health service, and to consider extending the duration of care for those who may not have transited by the end of the service period, which generally range between 1 and 3 years.

Future research should include the evaluation of other risk factors that can further refine the predictive accuracy of UHR states and may include the use of biological assays in risk calculations. We should also develop therapies that may prevent the onset of both psychotic and non-psychotic disorders and to improve the functional outcomes of these young persons.

## Author Contributions

CC responsible for data collection, data analysis and writing up of the manuscript. SV, MS, and EA preparation of the study protocol, data analysis, and writing of the manuscript. ST and LL preparation of the study protocol, writing of the manuscript.

### Conflict of Interest Statement

The authors declare that the research was conducted in the absence of any commercial or financial relationships that could be construed as a potential conflict of interest.

## References

[1] Global Burden of Disease Study 2013 Collaborators Global, regional, and national incidence, prevalence, and years lived with disability for 301 acute and chronic diseases and injuries in 188 countries, 1990–2013: a systematic analysis for the Global Burden of Disease Study 2013. Lancet, (2015) 386:743–800. 10.1016/S0140-6736(17)32154-2 26063472 PMC4561509

[2] Miller TJ McGlashan TH Rosen JL Somjee L Markovich PJ Stein K Prospective diagnosis of the initial prodrome for schizophrenia based on the Structure Interview for Prodromal Syndromes: Preliminary evidence of interrater reliability and predictive validity. Am J Psychiatry (2002) 159:863–5. 10.1176/appi.ajp.159.5.863 11986145

[3] Morrison A French P Walford L Lewis SW Kilcommons A Green J. Cognitive therapy for the prevention of psychosis in people at ultra-high risk: randomised controlled trial. Br J Psychiatry (2004) 185:291–7. 10.4324/9780203493465 15458988

[4] Haroun N Dunn L Harouna ASCK. Risk and protection in prodromal schizophrenia: ethical implications for clinical practice and future research. Schizophr Bull. (2006) 32:166–78. 10.1093/schbul/sbj007 16207892 PMC2632176

[5] Cannon T Cadenhead K Cornblatt B Woods S Addington J Walker E. Prediction of psychosis in youth at high clinical risk: a multisite longitudinal study in North America. Arch Gen Psychiatry (2008) 65:28–37. 10.1001/archgenpsychiatry.2007.3 18180426 PMC3065347

[6] Simon A Umbricht D. High remission rates from an initial ultra-high risk state for psychosis. Schizophr Res. (2010) 116:168–72. 10.1016/j.schres.2009.10.001 19854621

[7] Nelson B Hok Pan Y Wood S Lin A Spoliotacopoulos D Bruxner A Long-term follow-up of a group at ultra high risk (“Prodomal”) for psychosis - The PACE 400 study. JAMA Psychiatry (2013) 70:793–802. 10.1001/jamapsychiatry.2013.1270 23739772

[8] Ruhrmann S Schultze-Lutter F Salokangas R Heinimaa M Linszen D Dingemans P. Prediction of psychosis in adolescents and young adults at high risk: results from the prospective European prediction of psychosis study. Arch Gen Psychiatry (2010) 67:241–51. 10.1001/archgenpsychiatry.2009.206 20194824

[9] Yung A Yuen H Berger G Francey S Hung T Nelson B Declining transition rate in ultra high risk (prodromal) services: dilution or reduction of risk? Schizophr Bull. (2007) 33:673–81. 10.1093/schbul/sbm015 17404389 PMC2526154

[10] Addington J Cornblatt B Cadenhead K Cannon T McGlashan T Perkins D. At clinical high risk for psychosis: outcome for nonconverters. Am J Psychiatry (2011) 168:800–5. 10.1176/appi.ajp.2011.10081191 21498462 PMC3150607

[11] Simon AE Velthorst E Nieman DH Linszen D Umbricht D de Haan L. Ultra high-risk state for psychosis and non-transition: a systematic review. Schizophr Res. (2011) 132:8–17. 10.1016/j.schres.2011.07.002 21784618

[12] Lee J Rekhi G Mitter N Bong YL Kraus MS Lam M. The longitudinal youth at risk study (LYRIKS) - an asian UHR perspective. Schizophr Res. (2013) 151:279–83. 10.1016/j.schres.2013.09.025 24139196

[13] de Wit S Adolescents at ultrahigh risk for psychosis: long-term outcome of individuals who recover from their at-risk state. Eur Neuropsychopharmacol. (2014) 24:865–73. 10.1016/j.euroneuro.2014.02.008 24636460

[14] Lim J Rekhi G Rapisarda A Lam M Kraus M Keefe RS. Impact of psychiatric comorbidity in individuals at Ultra High Risk of psychosis - Findings from the Longitudinal Youth at Risk Study (LYRIKS). Schizophr Res. (2015) 164:8–14. 10.1016/j.schres.2015.03.007 25818728

[15] Rao S Santhathevi P Tay S Yuen S Poon L Lee H. Support for wellness achievement programme (SWAP): a service for individuals with at-risk mental state in singapore. Ann Acad Med. (2013) 42:552–5. 10.1111/eip.12176 24254247

[16] American Psychiatric Association Diagnostic and Statistical Manual of Mental Disorders. 4th ed. Washington, DC (1994).

[17] Goldman E Skodol A Lave T. Revising axis V for DSM-IV: a review of measures of social functioning. Am J Psychiatry (1992) 149:1148–56. 10.1176/ajp.149.9.1148 1386964

[18] Yung A Yuen H McGorry P Phillips L Kelly D Dell'olio M. Mapping the onset of psychosis: the comprehensive assessment of at-risk mental states. Aust N Z J Psychiatry (2005) 39:964–71. 10.1080/j.1440-1614.2005.01714.x 16343296

[19] Lam ML Hung SF Chen EY. Transition to Psychosis - 6-Month follow-up of a Chinese High-Risk Group in Hong Kong. Austr N Z J Psychiatry (2006) 40:414–20. 10.1080/j.1440-1614.2006.01817.x 16683967

[20] Yung A Stanford C Cosgrave E Killackey E Phillips L Nelson B. Testing the Ultra High Risk (prodromal) criteria for the prediction of psychosis in a clinical sample of young people. Schizophr Res. (2006) 84:57–66. 10.1016/j.schres.2006.03.014 16630707

[21] National Population and Talent Division P. M. Statistics, S. D., Affairs, M. O., Authority, I. A. (2014). 2014 Population in Brief. Singapore.

[22] Whitehorn D Brown J Richard J Rui Q Kopala L Multiple dimensions of recovery in early psychosis. Int Rev Psychiatry (2002) 14:273–83. 10.1080/0954026021000016914

[23] Fusar-Poli P Bonoldi I Yung A Borgwardt S Kempton M Valmaggia L. Predicting psychosis: meta-analysis of transition outcomes in individuals at high clinical risk. Arch Gen Psychiatry (2012) 69:220–9. 10.1001/archgenpsychiatry.2011.1472 22393215

[24] Fusar-Poli P Cappucciati M Borgwardt S Woods SW Addington J Nelson B. Heterogeneity of psychosis risk within individuals at clinical high risk: a meta-analytical stratification. JAMA Psychiatry (2016) 73:113–20. 10.1001/jamapsychiatry.2015.2324 26719911

[25] Schlosser D Jacobson S Chen Q Sugar C Niendam T Li G Recovery from an at-risk state: clinical and functional outcomes of putatively prodromal youth who do not develop psychosis. Schizophr Bull. (2012) 38:1225–33. 10.1093/schbul/sbr098 21825282 PMC3494042

[26] Johnstone E Ebmeier K Miller P Owens D Lawrie S. Predicting schizophrenia: findings from the Edinburgh High-Risk study. Br J Psychiatry (2005) 186:18–25. 10.1192/bjp.186.1.18 15630119

[27] Addington J Heinssen R. Prediction and prevention of psychosis in youth at clinical high risk. Ann Rev Clin Psychol. (2012) 8:269–89. 10.1146/annurev-clinpsy-032511-143146 22224837

[28] DeLisi LE. The significance of age of onset for schizophrenia. Schizophr Bull (1992) 18:209–215. 1377833 10.1093/schbul/18.2.209

[29] Rajji TK Ismail Z Mulsant BH. Age at onset and cognition in schizophrenia: meta-analysis. Br J Psychiatry (2009) 195:286–293. 10.1192/bjp.bp.108.060723 19794194

[30] Sham PC MacLean CJ Kendler KS. A typological model of schizophrenia based on age at onset, sex and familial morbidity. Acta Psychiatr Scand. (1994) 89:135–41. 10.1111/j.1600-0447.1994.tb01501.x 8178665

[31] Verma SK Subramaniam M Chong SA Kua EH. Substance abuse in schizophrenia. A Singapore perspective. Soc Psychiatry Psychiatr Epidemiol. (2002) 37:326–8. 10.1007/s00127-002-0553-8 12111024

[32] Central Narcotics Bureau Overview of Singapore's Drug Situation in 2017. Singapore (2018).

[33] McGorry P Nelson B Markulev C Yuen H Schäfer M Mossaheb N Effect of ω-3 polyunsaturated fatty acids in young people at ultrahigh risk for psychotic disorder. JAMA Psychiatry (2016) 74:19–27. 10.1001/jamapsychiatry.2016.2902 27893018

[34] Mason O Startup M Halpin S Schall U Conrad A Carr V. Risk factors for transition to first episode psychosis among individuals with “at risk mental states.” Schizophr Res. (2004) 71:227–37. 10.1016/j.schres.2004.04.006 15474894

[35] Velhorst E Nelson B Wiltink S de Haan L Wood S Lin A Transition to first episode psychosis in ultra high risk populations: does baseline functioning hold the key? Schizophr Res. (2013) 143:132–7. 10.1016/j.schres.2012.10.025 23182438

[36] Swanson J Swartz M Von Dorn R Elbogen E Wagner H Rosenheck R. A national study of violent behavior in persons with schizophrenia. Arch Gen Psychiatry (2006) 63:490–9. 10.1001/archpsyc.63.5.490 16651506

[37] Moulin V Palix J Golay P Dumais A Gholamrezaee M Azzola A. Violent behaviour in early psychosis patients: can we identify clinical risk profiles? Early Interv Psychiatry (2017). [Epub ahead of print]. 10.1111/eip.12512 29143486

[38] Moulin V Golay P Palix J Baumann PS Gholamrezaee MM Azzola A. Impulsivity in early psychosis: A complex link with violent behaviour and a target for intervention. Eur Psychiatry (2018) 49:30–6. 10.1016/j.eurpsy.2017.12.003 29353178

[39] Nelson B Yuen K Yung A. Ultra high risk (UHR) for psychosis criteria: are there different levels of risk for transition to psychosis? Schizophr Res. (2011) 125:62–8. 10.1016/j.schres.2010.10.017 21074975

[40] Brandizzi M Valmaggia L Byrne M Jones C Iewgbu N Badger S. Predictors of functional outcome in individuals at high clinical risk for psychosis at six years follow-up. J Psychiatric Res. (2015) 65:115–23. 10.1016/j.jpsychires.2015.03.005 25837413

[41] Government of Singapore (2001). Compulsory Education Act (Chapter 51).

